# Lignin-AuNPs liquid marble for remotely-controllable detection of Pb^2+^

**DOI:** 10.1038/srep38164

**Published:** 2016-12-02

**Authors:** Guocheng Han, Xiaoying Wang, Jonathan Hamel, Hongli Zhu, Runcang Sun

**Affiliations:** 1 State Key Laboratory of Pulp & Paper Engineering, South China University of Technology, Guangzhou, 510640, China; 2 Department of Mechanical and Industrial Engineering, Northeastern University, Boston, MA, 02115, United States

## Abstract

This work reported the green and facile fabrication of a versatile lignin-AuNP composite, which was readily and remotely encapsulated to form novel liquid marbles. The marbles can stay suspended in water, and show excellent photothermal conversion properties, as well as visual detection and adsorption towards Pb^2+^. More importantly, the marbles can simultaneously remotely detect and adsorb Pb^2+^ via co-precipitation by simply controlling the near infrared (NIR) irradiation. It is believed that the remotely-controllable NIR-responsive lignin-AuNPs liquid marble can be used in Pb^2+^-related reactions. The liquid marble can be placed in the system at the very beginning of the reaction and stably stays on the surface until the reaction has ended. After reacting, upon remote NIR irradiation, the liquid marble bursts to adsorb Pb^2+^, and the residual Pb^2+^ can be collected. This facile manipulation strategy does not use complicated nanostructures or sophisticated equipment, so it has potential applications for channel-free microfluidics, smart microreactors, microengines, and so on.

Gold nanoparticles with controlled size, morphology, and good dispersibility are attracting great attention due to their special properties^1^,^2^. Several noteworthy studies have illustrated multifunctional Au Nanoparticle-based materials for independent sensing, photothermal conversion^3^, simultaneous detection and imaging^4^, and targeted chemo-photothermal treatments^5^. Layer-by-layer assembly^3^, core-shell structure^6^, or ligand functionality is applied^7^, showing the versatility of AuNPs. In other areas of nanotechnology, the liquid marble technique has been explored in multiple applications such as chemical sensors and miniature reactors^8^. The liquid marble system can reduce the use of highly toxic, reactive, or costly reagents in hazardous reactions, and keep operators away from dangerous or inaccessible environments when the liquid marble is remotely-controllable^9^. Therefore, it is an interesting way to present the versatility of AuNPs via a facile liquid marble stragegy, but so far, few studies have focused on this.

In addition, due to biological or environmental-related demands, there is an increasing emphasis on synthesizing AuNPs with nontoxic chemicals, and safer solvents and reaction conditions^10^. Over the last decade, several eco-friendly procedures have been developed to synthesize AuNPs based on natural reducing sugars, plant extracts, and intracellular biosynthesis^11^,^12^. However, many of these feedstocks are not abundant in nature, so additional extraction or enrichment processes are required^13^. These procedures are therefore identified as cost-effective but less “green”. Considering the idea of sustainable development and economic viability, a “greener” feedstock candidate should be proposed for further industrial production of AuNPs. Lignin, a biopolymer derived from trees and crops, is the second-most abundant biopolymer in nature and the main by-product of the paper industry. Historically, the majority of lignin was burned as fuel or used in low-value applications such as activated carbon, dispersants, and adhesive agents^14^. Currently, scientists have noticed that the reducing groups and 3D spatial structure of lignin provide the possibility for green synthesis of metal nanoparticles, thus avoiding hazardous chemicals and unnecessary modification^15^,^16^,^17^.

In this study, we first investigate the green synthesis of AuNPs based on lignin. To reveal the versatility of lignin-AuNPs composites, the photothermal activity and visual detection/adsorption towards Pb^2+^ were studied. Furthermore, for the first time, we fabricated NIR-responsive lignin-AuNPs liquid marbles as remotely-controllable miniature reactors for Pb^2+^ detection and adsorption (seen in Fig. 1). Finally, the differences between lignin-AuNPs liquid marbles and conventional liquid marbles as well as the suggested mechanism of the NIR-responsive process of lignin-AuNPs liquid marbles are discussed.

## Results and Discussion

### Structure and morphology of lignin-AuNPs composites

**Figure 2:**
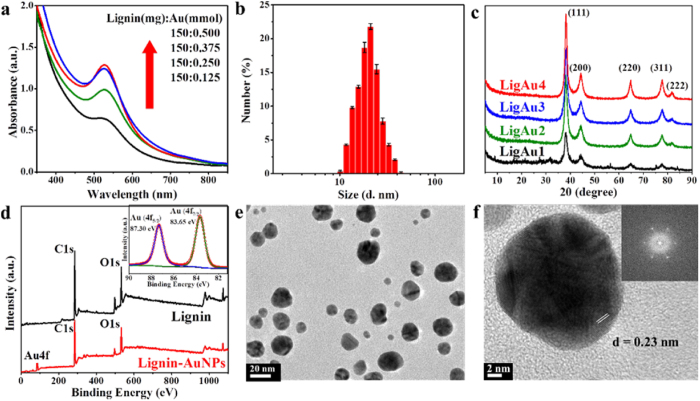
(**a**) UV-Vis spectra of lignin-AuNPs composites; (**b**) size distribution analysis of lignin-AuNPs composites synthesized under optimal condition, the *x*-axis is in log scale; (**c**) XRD patterns of the lignin-AuNPs composites with different Au ratios; (**d**) XPS spectra of lignin and lignin-AuNPs composite (inset: high-resolution XPS spectra of Au 4 f of lignin-AuNPs composite); (**e**) TEM image of lignin-AuNPs composites synthesized under optimal condition and (**f**) HRTEM image of single AuNP (inset: Fast Fourier Transform (FFT) of AuNP).

Figure 2(a) shows the UV-Vis spectra of lignin-AuNPs composites synthesized at different ratios of lignin to HAuCl_4_. A feature typical of surface plasmon resonance (SPR) at around 520 nm with a single peak is observed, indicating the formation of AuNPs^18^. As the ratio of lignin to HAuCl_4_ was increased, the shape of the plasmon peak became more symmetrical, suggesting the formation of spherical and monodispersed AuNPs^19^. It was found that the optimal mass ratio of lignin to HAuCl_4_ was 150 mg to 0.375 mmol. In addition, the UV-Vis spectra, Au elemental analysis and TEM images of AuNPs synthesized under different reaction temperatures and times are illustrated in Fig. S1, Table S1 and Fig. S2, respectively. These data show that the formation amount of AuNPs was positively related to reaction temperature and time. Therefore, the optimum condition for green synthesis of AuNPs with lignin was a ratio of 150 mg of lignin to 0.375 mmol of HAuCl_4_, a reaction temperature of 80 °C, and an irradiation time of 60 min. Under optimal conditions, a narrow distribution in the particle size (about 20 nm) can be obtained (as shown in Fig. 2(b)), which is in agreement with the UV-Vis and TEM results.

The XRD patterns of the lignin-AuNPs composites with different gold contents are shown in Fig. 2(c). Five intense reflection peaks at 2*θ* = 38.2°, 44.4°, 64.6°, 77.6°, and 81.7° correspond to the (111), (200), (220), (311) and (222) facets, respectively. These peaks matched with JCPDS (No. 04-0784), revealing the composition of pure crystalline gold^20^. The ratio between the intensity of the (200) and (111) diffraction peaks was 0.29, which was lower than the conventional bulk intensity ratio (0.52), suggesting that the (111) plane was the predominant orientation and the AuNPs were mostly spherical^21^.

The XPS spectra in Fig. 2(d) show that besides C and O elements in lignin, there was also elemental Au contained in lignin-AuNPs composites. The high-resolution XPS spectrum of Au 4 f on lignin-AuNPs composites is shown in the inset of Fig. 2(d). The Au 4 f spectrum can be divided into two individual peaks at 83.6 eV and 87.3 eV, which are attributed to Au 4 f_7/2_ and Au 4f_5/2_ binding energies. The Au 4f doublet matched with fixed spectroscopic parameters, such as spin-orbital separation (~3.7 eV) and the 4f_5/2_:4f_7/2_ branching ratio of 0.75^22^. These results indicate the crystalline nature of AuNPs. In this case, a small shift of binding energies was observed, compared to the pure metallic Au 4f_7/2_ of 84.0 eV^23^. This may be attributed to the interaction between AuNPs and the stabilizing agent lignin^24^.

Figure 2(e) shows the TEM image of lignin-AuNPs composites synthesized under optimal conditions, which were mostly spherical and well-dispersed with a relatively narrow size distribution. From the HRTEM image in Fig. 2(f), it can be seen that the AuNP was in a crystalline state with spherical morphology and a diameter of ~20 nm. The interplanar d-spacing calculated from the lattice-resolved image of the spherical AuNPs was 0.23 nm, which matches with that of the (111) plane of the typical Au metal (JCPDS file No. 04-0784)^20^. The Fast Fourier Transform (FFT) indicates the monocrystalline nature of the formed AuNPs. The result is in agreement with the XPS spectra shown in Fig. 2(d).

### Photothermal activities of lignin-AuNPs composites paper

**Figure 3:**
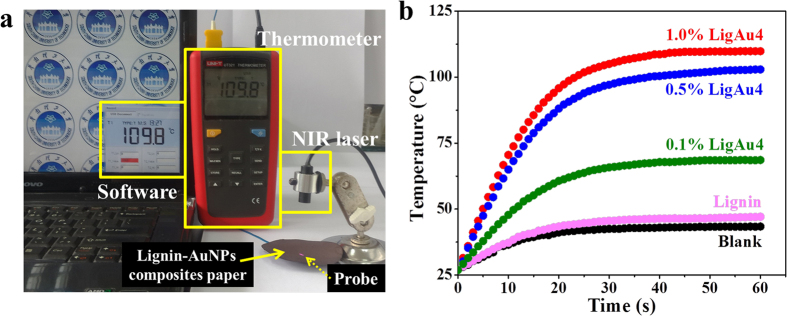
(**a**) Optical photo of the experimental setup; (**b**) Temperature rise trace of lignin-AuNPs composite papers with different dosage of lignin-AuNPs.

Due to the localized plasmon resonances of AuNPs, as well as the improvement effect of the surrounding materials, the adsorbed light would mostly convert to heat by AuNPs. This behavior is known as photothermal conversion effect^3^,^25^. To explore the photothermal activities, lignin-AuNPs composites were added into filter paper. As shown in Fig. S3, paper immersed with 0.5% lignin looked yellowish (the natural color of lignin), and the color of paper immersed in different concentrations and content of lignin-AuNPs composites turned from grey to dark brown. The experimental apparatus in Fig. 3(a) shows an 808 nm infrared diode laser with a power density of 4.2 W/cm^2^ as the light source to irradiate the lignin-AuNPs composites paper. The heat generated by the lignin-AuNPs composites was detected by a thermal probe directly under the paper. The temperature variation of the thermal probe with the prolonged irradiation time was recorded and plotted by a thermometer and computer software, and the average heating rate in a period of 60 s was calculated. The results are shown in Fig. 3(b), Table S2 and Fig. S4. The blank sample showed little increment in temperature. The lignin-only paper performed slightly better than the blank sample, which may be due to the photosensitive effect of phytochrome within lignin^26^. It can clearly be seen from Fig. 3(b) that paper immersed with lignin-AuNPs composites exhibited a quicker temperature increment than the samples without AuNPs, which is an indication of the photothermal conversion functionality generated by AuNPs. Furthermore, as the concentration of lignin-AuNP composites increased, a higher heating rate and final temperature was observed, as seen in Fig. 3(b). When the concentration of the immersion solution increased from 0.1% to 1.0%, the ultimate temperature increased and reached a maximum of about 110 °C, with a maximum heating rate of 1.830 °C/s. On the other hand, the ultimate temperature increased as the Au content of lignin-AuNPs composites increased, as shown in Table S2. Hence, the photothermal activity of lignin-AuNPs composites can be tuned by simply changing the Au content or immersion concentration of lignin-AuNP composites. The rapid temperature rise and high final temperature were caused by the photothermal conversion effect of the lignin-AuNP composites, which shows their potential in applications such as photothermal therapy or NIR-response.

### Simultaneous naked-eye detection and adsorption for Pb^2+^

**Figure 4:**
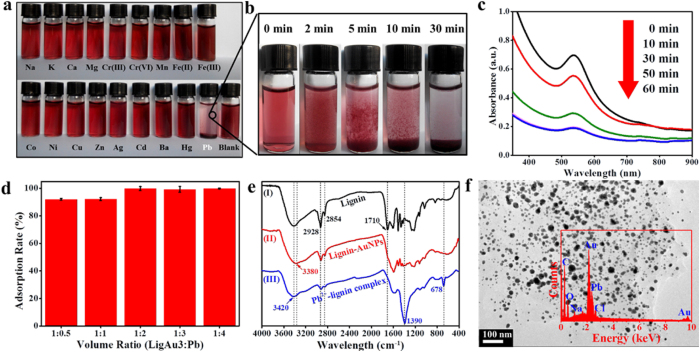
(**a**) Images of solutions containing LigAu3 and various metal ions in a volume ratio of 1:2; (**b**) Images depicting the change of the LigAu3/Pb^2+^ system; (**c**) UV-V is spectra of LigAu3/Pb^2+^ solution with prolonged time; (**d**) Adsorption rates for Pb^2+^ by LigAu3/Pb^2+^ solutions in different volume ratios; (**e**) FT-IR spectra of (I) lignin, (II) lignin-AuNPs composites and (III) precipitate obtained from the LigAu3/Pb^2+^ system in a ratio of 1:2; (**f**) TEM image of the precipitate in the LigAu3/Pb^2+^ system (inset: corresponding EDS profile of the precipitate).

Unlike previous studies on the AuNP probes for the detection of Pb^2+ ^27, the prepared lignin-AuNP composites showed simultaneous naked-eye detection and adsorption for Pb^2+^, which was not reported before. As Fig. 4(a) shows, among various metal ions only the Pb^2+^ solution (10^−3^ M) exhibited color fading and precipitation after addition of lignin-AuNP composites (10 mg/mL) in a volume ratio of 1:2 (LigAu3:Pb) within 5 min. This can be visualized with the naked eye. Moreover, the change of the LigAu3/Pb^2+^ system was further monitored by a digital camera (Fig. 4(b)) and UV-Vis spectra (Fig. 4(c)). In Fig. 4(b), the mixture of lignin-AuNP composites and Pb^2+^ was a red color at the very beginning due to the natural color of AuNPs. About 2 minutes later, the solution became cloudy, teeming with micro floccule. After another few minutes elapsed, the micro floccule gradually aggregated and deposited. 10 minutes later, plenty of red precipitation could clearly be seen in the bottom of the glassware and the color of solution had become lighter. Finally, after 30 minutes of standing, most micro floccule had deposited and the supernatant became almost colorless and clear. The UV-Vis spectra in Fig. 4(c) showed that in the LigAu3/Pb^2+^ system, the featured SPR of AuNPs around 520 nm gradually weakened over time, which is in accordance with the results monitored by the naked eye. These results indicate that the lignin-AuNP composites were precipitated after mixing with Pb^2+^. An effective removal of Pb^2+^ from the LigAu3/Pb^2+^ system along with the precipitation was discovered using AAS measurement. After standing and centrifugation, the adsorption rate for the Pb^2+^ in LigAu3/Pb^2+^ (ratio of 1:2) system reached 99%. When varying the volume ratios of LigAu3 composites and Pb^2+^ solution from 1:0.5 to 1:4 (Fig. 4(d)), the removal rates remained over 90%, indicating that lignin-AuNPs composites have excellent properties for the removal of Pb^2+^.

The FT-IR spectra of lignin before and after the reducing reaction are shown in Fig. 4(e), spectrum I and II. After the redox reaction, the bands at 2928 cm^−1^ and 2854 cm^−1^ corresponded to stretching vibrations of −CH_2_− and −CH_3_ in lignin were diminished or disappeared entirely (spectrum II), indicating that the alkyl chains of lignin may be cut off. Compared with spectrum I, the C=O band of ester at 1710 cm^−1^ disappeared in spectrum II, which may imply that the non-conjugated C=O units in ester transformed into carboxyl after the formation of AuNPs. This is therefore evidence of the reducing ability of lignin^15^. The suggested reaction process of lignin for AuNP synthesis is shown in Fig. S5.

The Pb^2+^ binding mechanism was investigated by FT-IR, scanning electron microscopy (SEM) and energy dispersive spectroscopy (EDS). The spectrum II and III in Fig. 4(e) show the differences before and after the adsorption of Pb^2+^. The band at 3380 cm^−1^ corresponding to −OH was shifted to 3420 cm^−1^ in spectrum III, which may be a result of the interaction between –OH of lignin and Pb^2+^. The C=O stretching vibration at 1390 cm^−1^ was observed in spectrum III after the adsorption, which can be ascribed to the carboxyl of aryl acid^15^. The aryl acid may be separated from lignin-AuNPs composites when chelated with Pb^2+^. In spectrum III, the band of the benzene ring at 678 cm^−1^ appeared after the adsorption of Pb^2+^, which may be because of the complexation between Pb^2+^ and the benzene ring of lignin structure. Thus, the property of lignin-AuNPs composites to adsorb Pb^2+^ can be ascribed to the hydroxyl groups and carbonyl groups on lignin as a strong Lewis base^28^. The phenolic units on lignin appeared to have stronger affinity towards Pb^2+^ than Au and other metal ions^29^, forming a Pb^2+^-lignin chelation complex. The Pb^2+^-lignin complex may be poorly soluble in aqueous solution and probably destabilized the lignin-AuNPs composites, leading to precipitation of the Pb^2+^-lignin complex together with aggregated AuNPs. The TEM image in Fig. 4(f) showed that the precipitate was a complex of lignin and aggregated AuNPs, spreading out hundreds of nanometers in size. And the corresponding EDS profile not only showed carbon and oxygen signals from the biomolecules of lignin in the precipitate, but also Au and Pb signals, indicating the adsorption of Pb^2+^ on lignin-AuNPs composites. Therefore, a conclusion can be drawn that a remarkable property of naked-eye detection and adsorption for Pb^2+^ by lignin-AuNPs composites was achieved simultaneously, presenting a promising alternative for contaminant detection and adsorption.

### Properties of multifunctional lignin-AuNPs liquid marbles

**Figure 5:**
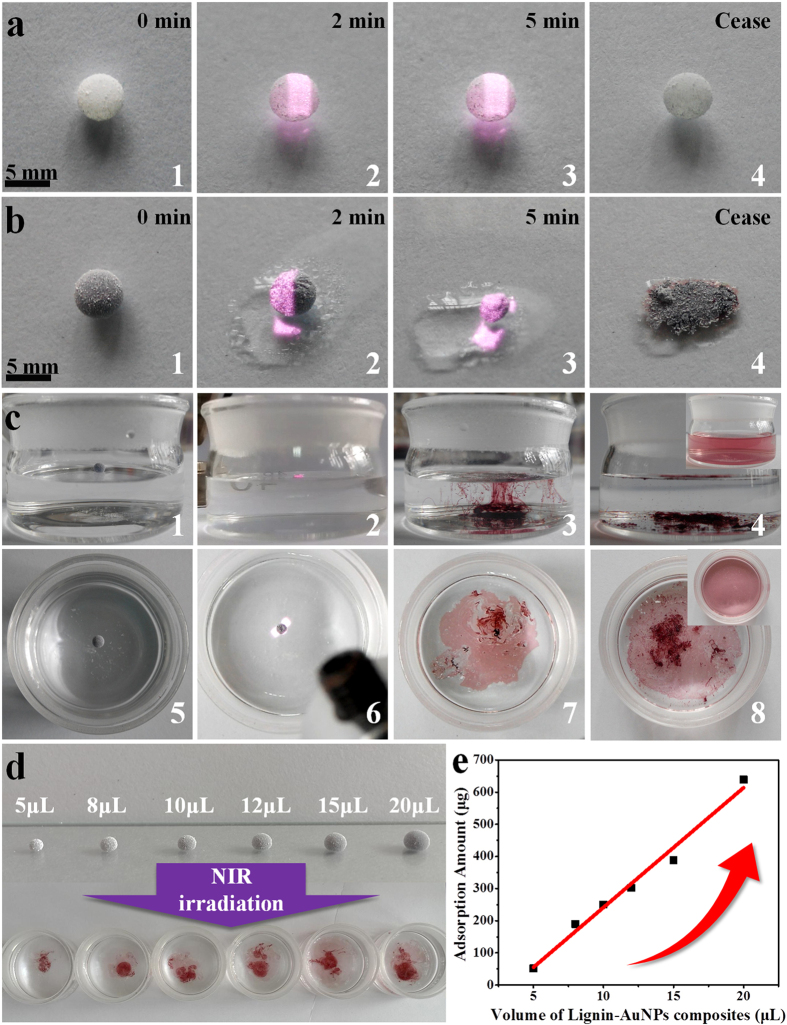
(**a**) Water-based liquid marble irradiated under near-infrared on glass substrate; (**b**) Lignin-AuNPs composites liquid marble irradiated on glass substrate; (**c**) Front view and top view images of a lignin-AuNPs liquid marble irradiated in Pb^2+^ solution (insets: final state of the same liquid marble irradiated on pure water); (**d**) liquid marbles fabricated using different volume of lignin-AuNPs composites from 5 to 20 μL, and bursting on 10 mL of Pb solution (10^−3^ M) after NIR irradiation; (**e**) adsorption amount of Pb^2+^ by lignin-AuNPs liquid marbles with different volumes ranging from 5 to 20 μL.

To combine both the photothermal conversion and the Pb^2+^ detection/adsorption properties of the lignin-AuNPs composites for practical applications, a facile liquid marble strategy was used for the first time. First, the NIR-responsive activity of the lignin-AuNPs liquid marbles was investigated. As seen in Fig. 5(a), a blank liquid marble composed of 15 μL of water showed non-responsive properties to NIR irradiation. The shape and volume of the blank marble changed little during a 5 minutes irradiation period (Fig. 5(a), images 1~4). The liquid marble made from 15 μL of lignin-AuNPs composites appeared purple and had a diameter of about 5 mm (Fig. 5(b), image 1). The lignin-AuNPs marble can stably stand on a glass substrate. However, when subjected to NIR irradiation, the water in lignin-AuNPs composites droplet permeated through the gaps of the PVDF shell due to stronger photothermal conversion activity of lignin-AuNPs composites inside that hemisphere (seen in Fig. 5(b), image 2). Moreover, the liquid marble showed significant reduction in size, became non-uniform after 5 minutes’ irradiation, and most water inside the marble was observed to leak out (seen in Fig. 5(b), image 3). Soon after ceasing the irradiation, the structure of the marble collapsed, and the concentrated lignin-AuNP composites bled from inside (seen in Fig. 5(b), image 4).

Furthermore, the adsorption property of lignin-AuNPs liquid marbles towards Pb^2+^ under irradiation was studied, as shown in Fig. 5(c) from the top view and front view, also seen in Movie S1. In Fig. 5(c), image 1 and 5, the liquid marble containing 15 μL of lignin-AuNP composites was allowed to float stably on 10 mL Pb^2+^ solution before NIR irradiation. During the irradiation, the marble shrank in size and became flat (seen in Fig. 5(c), image 2). Gradually, the liquid marble collapsed and then suddenly ruptured. The lignin-AuNPs composites spread on the solution surface and diffused into the solution, immediately forming some red-color floccules (seen in Fig. 5(c), image 3 and 7). According to Section 3.3, the floccules were Pb^2+^-lignin complex along with aggregated AuNPs. Lastly, most floccules precipitated and the solution remained colorless (seen in Fig. 5(c), image 4 and 8), indicating few AuNPs were dissolved into water. This is in comparison to a red-color AuNPs solution resulting from the rupture of lignin-AuNP liquid marble in pure water (see the insets in Fig. 5(c), image 4 and 8). The whole experiment lasted a few minutes and was recorded in Movie S1. The liquid marble was also put in the CuSO_4_, ZnSO_4_, Cd(NO_3_)_2_ solution, but after NIR irradiation, the liquid marble collapsed and solved into the solution with no precipitation, which is in agreement with the result from lignin-AuNP composites. In addition, when the liquid marbles were fabricated with 5 to 20 μL of lignin-AuNP composites (Fig. 5(d)), the sizes increased, and the liquid marbles are all free-standing and stable on either glass substrates or an aqueous surface. NIR irradiation of the liquid marbles on the Pb solution has the same effect as described above. When precipitation is complete, the adsorption amounts of Pb^2+^ by lignin-AuNPs composite liquid marbles with different volumes were determined by AAS, as shown in Fig. 5(e). The results demonstrate that larger volumes of lignin-AuNPs adsorb more Pb^2+^. The liquid marble with 20 μL of lignin-AuNPs composites could adsorb over 600 μg of Pb^2+^. The linear relationship between the volume of lignin-AuNPs composites and adsorption amount of Pb^2+^ implies the possibility of controllable adsorption for practical requirement. After the Pb^2+^ adsorption, the LigAu3/Pb^2+^ complex can be incinerated to remove the organic component. The Au and Pb can be easily separated and recycled via acid treatment. Therefore the Au can be recycled and reused.

**Figure 6:**
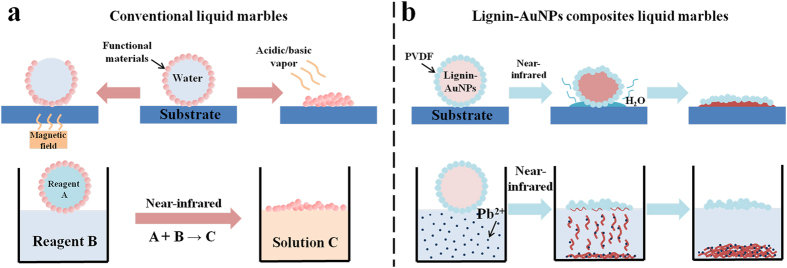
(**a**) Mechanism of some conventional liquid marbles. (**b**) NIR-responsive lignin-AuNP liquid marbles and the schematic process for Pb^2+^ adsorption.

Figure 6 shows the differences between conventional liquid marbles and the presented lignin-AuNPs liquid marble, as well as the suggested mechanism of the NIR-responsive process in this study. Conventional liquid marbles are usually composed with a shell of hydrophobic functional materials, such as highly hydrophobic Fe_3_O_4_ nanoparticles^30^, multifunctional Janus particles^31^ or carbon nanotubes^32^, which are responsive to the magnetic field, acidic/basic vapors, or NIR. The containers of conventional liquid marbles are usually water or reagents with no response to ambient conditions. Interestingly, in this study we adopt a new strategy by placing the versatile AuNP composites inside the liquid marble. The liquid marbles are easily prepared by covering lignin-AuNP composites with commercial PVDF, which is responsive to NIR, allowing it to be remotely controlled. When the NIR irradiation began, the lignin-AuNPs composites inside the liquid marble adsorbed the irradiation through the loose PVDF shell and locally converted NIR energy into heat. The water inside the lignin-AuNPs composite droplet gradually evaporated and permeated through the gaps of the PVDF shell due to the heating^32^, resulting in a reduction in size and a liquid marble with a wrinkled surface. When most of the lignin-AuNP composites inside the liquid marble were gone, the structure became unstable and was on the verge of collapse. A slight change of ambient conditions such as pressure or temperature would trigger bursting of the condensed lignin-AuNP composites. As a result, the lignin-AuNPs passed through the gas-liquid interface and dispersed in the aqueous solution, and then contaminants like Pb^2+^ were adsorbed and removed from the aqueous system.

## Conclusions

We used lignin as green feedstock, and reducing and stabilizing agent to synthesize the AuNPs and then fabricated a remotely-controllable NIR-responsive lignin-AuNPs liquid marble. The marble combined the photothermal conversion and Pb^2+^ detection/adsorption properties of AuNPs in a new way, so that a remote-controlled recycling process could be demonstrated. The study not only provides a green way of synthesizing gold nanoparticles based on recovered lignin from pulping processes, but also proposes an emerging strategy of utilizing multifunctional AuNPs.

## Experimental

### Synthesis of lignin-AuNP composites

First, a 2% (w/v) lignin aqueous solution was obtained by dissolving 0.150 g of recovered lignin in 1 wt. % NaOH solution and stirring for 30 min. Then a predetermined amount of HAuCl_4_·3H_2_O was dissolved in a flask with deionized water and placed into a microwave synthesis system (800 W). The lignin solution is slowly dropped into the HAuCl_4_ solution and reacted under microwave radiation at a designed temperature and duration. During the reaction, the formation of AuNPs was observed by the change of the solution color from dark brown to dark red. After the reaction the collected products were dialyzed to remove Au^3+^ and neutralize the pH. Finally, the lignin-AuNP composites are obtained after lyophilization at −40 °C. Table S1 shows a series of lignin-AuNP composites synthesized under different conditions.

### Characterization of lignin-AuNP composites

UV-Vis spectra of lignin-AuNPs composites were collected on a UV-1800 spectrophotometer (Shimadzu, Japan). Size distribution analysis of lignin-AuNP composites were carried out on a Malvern 3000HSA analyzer (Malvern, England). JEM-2010HR transmission electron microscopy (TEM) (JEOL, Japan) was used to observe the microstructure of AuNPs. The crystal structure of AuNPs was determined by D8 Advance X-ray diffractometer (XRD) (Bruker, Germany). X-ray photoelectron spectroscopy (XPS) spectra of the samples were recorded by Kratos AXIS Ultra DLD (Kratos, UK). Fourier transform infrared (FT-IR) spectra were measured on a Vector 33 spectrophotometer (Bruker, Germany) by KBr pellet method.

### Properties of lignin-AuNP composites

#### Photothermal activity of lignin-AuNP composites

The photothermal conversion effect of lignin-AuNP composites was investigated under the support of commercial filter paper. The filter paper was immersed into different concentrations of lignin-AuNP composite solution for 2 h, and dried at 50 °C in an oven. An 808 nm near infrared (NIR) diode laser (Changchun New Industries Optoelectronics Technology Co., Ltd.) with a power density of 4.2 W/cm^2^ was used as the light source to irradiate the lignin-AuNP composite paper. The heat generated by the lignin-AuNP composites was detected by a thermal probe and thermometer (UNI-T 1310, UNI-T Electronic Corp.).

#### Simultaneous visual detection and adsorption of Pb^2+^

A well-dispersed lignin-AuNP composite solution with concentration of 0.1 mg/mL was prepared. An aqueous solution containing Pb^2+^ (Pb(NO_3_)_2_, 10^−3^ mol/L) was mixed with lignin-AuNP composite solution at a specific volume ratio. Other metal ion solutions (NaCl, KCl, CaCl_2_, MgCl_2_, Cr(NO_3_)_3_, K_2_Cr_2_O_7_, MnCl_2_, FeSO_4_, FeCl_3_, Co(NO_3_)_2_, Ni(NO_3_)_2_, CuSO_4_, ZnSO_4_, AgNO_3_, Cd(NO_3_)_2_, BaCl_2_, HgCl_2_; 10^−3^ mol/L) were also mixed with lignin-AuNP composite solution for contrast. Afterwards, the change of the solution colors were observed using the naked eye and monitored by UV-1800 UV-Vis spectrophotometer (Shimadzu, Japan). After standing for one hour, the mixture was centrifuged and the adsorption percentages of lignin-AuNP composites for Pb^2+^ were measured by atomic absorption spectrometry (AAS) (Jena, Germany).

### The application of lignin-AuNP composites via liquid marbles strategy

The lignin-AuNP liquid marbles were easily fabricated by rolling lignin-AuNP composite droplets on a powder bed of polyvinylidene fluoride (PVDF) until they were encapsulated completely. Typically this occurred in less than 10 seconds. The sizes of the liquid marbles were controllable by varying the droplet volume. The liquid marbles were placed on a glass substrate or water surface. The thermal response was actuated under 808 nm NIR irradiation with a power density of 4.2 W/cm^2^.

## Additional Information

**How to cite this article**: Han, G. *et al*. Lignin-AuNPs liquid marble for remotely-controllable detection of Pb^2+^. *Sci. Rep.* **6**, 38164; doi: 10.1038/srep38164 (2016).

**Publisher's note:** Springer Nature remains neutral with regard to jurisdictional claims in published maps and institutional affiliations.

## Supplementary Material

Supplementary Information

Supplementary Video S1

## Figures and Tables

**Figure 1:**
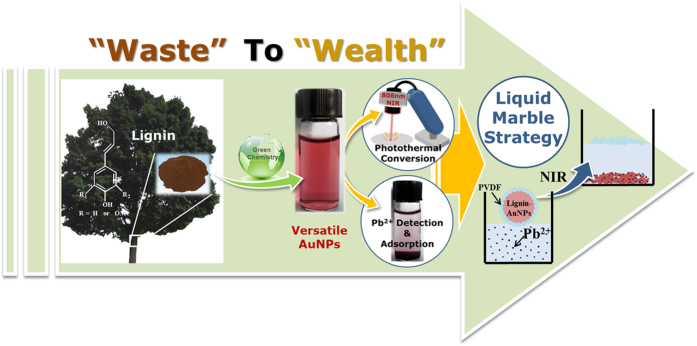
Illustration of this study: lignin is used to synthesize versatile AuNPs, and a novel liquid marble strategy was employed to combine the photothermal conversion and simultaneous Pb^2+^ detection and adsorption properties of the lignin-AuNPs composites, resulting in NIR-responsive lignin-AuNPs liquid marbles.
