# Supplementary material for: Lignin-AuNPs liquid marble for remotely-controllable detection of Pb2+
Source: Sci Rep. 2016 Dec 2;6:38164. doi: 10.1038/srep38164 (PMC5133569; doi:10.1038/srep38164)
Supplement: Supplementary Information [file srep38164-s1.doc]

**Supporting Information**

**Lignin-AuNPs liquid marble for remotely-controllable detection of Pb^2+^**

Guocheng Han^a^, Xiaoying Wang^a,*^, Jonathan Hamel^b^, Hongli Zhu^b,*^, Runcang Sun^a^

^a^ State Key Laboratory of Pulp & Paper Engineering, South China University of Technology, Guangzhou, 510640, China

^b^ Department of Mechanical and Industrial Engineering, Northeastern University, Boston, MA, 02115, United States

^*^ Corresponding author: Xiaoying Wang, E-mail: [xyw@scut.edu.cn](mailto:xyw@scut.edu.cn); Hongli Zhu, E-mail: [h.zhu@neu.edu](mailto:h.zhu@neu.edu)

***Materials and apparatus***

Lignin recovered from wheat straw by the soda pulping process was obtained from the State Key Laboratory of Pulp & Paper Engineering (Guangzhou, China) for this study. Gold (III) chloride hydrate (HAuCl_4_·3H_2_O) was purchased from Aladdin Industrial Inc. (Shanghai, China). All other chemical reagents and solvents were of analytical grade.

An XH-100A microwave synthesis system was purchased from Beijing XiangHu Sci.-Tech. Dept. Co., Ltd. (Beijing, China). The microwave power ranged from 100 to 1000 W, and the highest reaction temperature is 300 °C.

**Table S1.** Lignin-AuNP composites and Au elemental analysis obtained from different reaction conditions

| **Samples** | **Lignin:HAuCl_4_**  **(mg:mmol)** | **Reaction**  **temperature (°C)** | **Reaction time (min)** | **Au content (%)** |
| --- | --- | --- | --- | --- |
| LigAu1 | 150:0.125 | 80 | 60 | 9.42 |
| LigAu2 | 150:0.250 | 80 | 60 | 19.88 |
| LigAu3 | 150:0.375 | 80 | 60 | 24.50 |
| LigAu4 | 150:0.500 | 80 | 60 | 25.21 |
| LigAu5 | 150:0.375 | 60 | 60 | 15.11 |
| LigAu6 | 150:0.375 | 70 | 60 | 19.06 |
| LigAu3 | 150:0.375 | 80 | 60 | 24.50 |
| LigAu7 | 150:0.375 | 90 | 60 | 27.73 |
| LigAu9 | 150:0.375 | 80 | 10 | 21.00 |
| LigAu10 | 150:0.375 | 80 | 20 | 22.21 |
| LigAu12 | 150:0.375 | 80 | 40 | 23.65 |
| LigAu3 | 150:0.375 | 80 | 60 | 24.50 |
| LigAu15 | 150:0.375 | 80 | 80 | 26.85 |

**Table S2.** Photothermal conversion effect of different lignin-AuNP composite papers

| **Samples** | **Concentration of immersion solution (wt. %)** | **Ultimate Temperature (°C)** | **Heating rate (°C/s)** |
| --- | --- | --- | --- |
| **Blank** | - | 43.0 | 0.717 |
| **Lignin** | 0.5 | 47.0 | 0.783 |
| **LigAu1** | 0.5 | 77.1 | 1.285 |
| **LigAu3** | 0.5 | 95.9 | 1.598 |
| **LigAu4** | 0.5 | 102.9 | 1.715 |
| **LigAu4** | 1.0 | 109.8 | 1.830 |
| **LigAu4** | 0.1 | 68.8 | 1.147 |


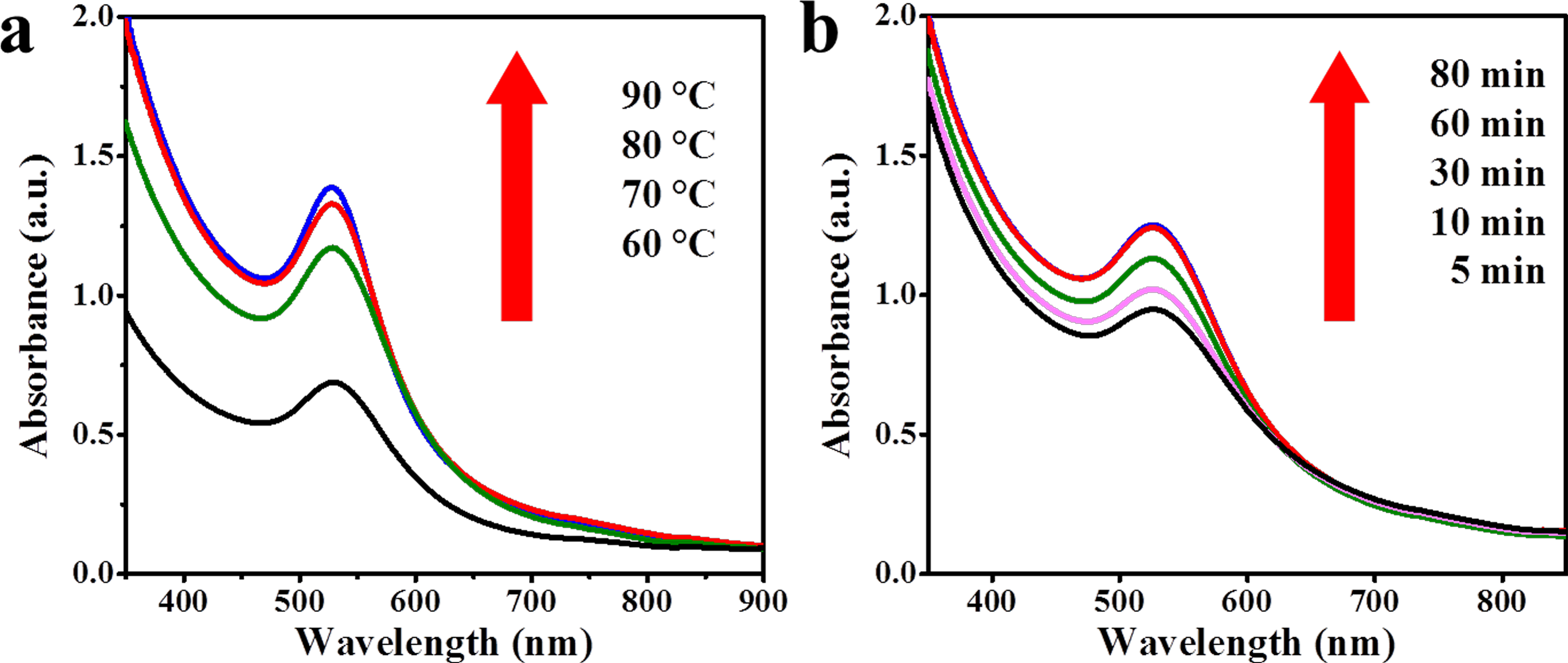


**Fig. S1**. UV-Vis spectra of lignin-AuNPs composites: (a) under different temperatures and (b) under different times

As illustrated in the UV-Vis spectra in Fig. S1(a), the absorbance of the spectrum increased with the temperature increase from 60 °C to 90 °C, which means the formation amount of AuNPs was positively related to the reaction temperature. When the temperature was increased from 60 °C to 70 °C, there was a large increase in absorbance, suggesting an accelerated formation of AuNPs. But when the temperature was increased further, higher than 80 °C, the increase of absorbance became smaller, which may be because the reaction had reached equilibrium. Higher reaction temperatures led to narrower plasmon peaks. It can be observed that composites synthesized at 80 °C or higher had a narrow full width at half-maximum (FWHM), indicating a narrow distribution in the particle size [1]. Considering the energy savings, a reaction temperature at 80 °C was preferred.

The changes of UV-Vis spectra with prolonged reaction time are shown in Fig. S1(b). The figure shows that the absorbance of the spectrum increased with prolonged irradiation time, revealing an increasing amount of AuNPs. This result shows that the reaction time had a beneficial effect on the increasing amount of AuNPs. Fig. S1(b) demonstrates that when the irradiation time exceeded 60 min, the plasmon intensity barely changed, indicating the reaction was near completion. As a result, the optimal reaction time was chosen to be 60 min.

**
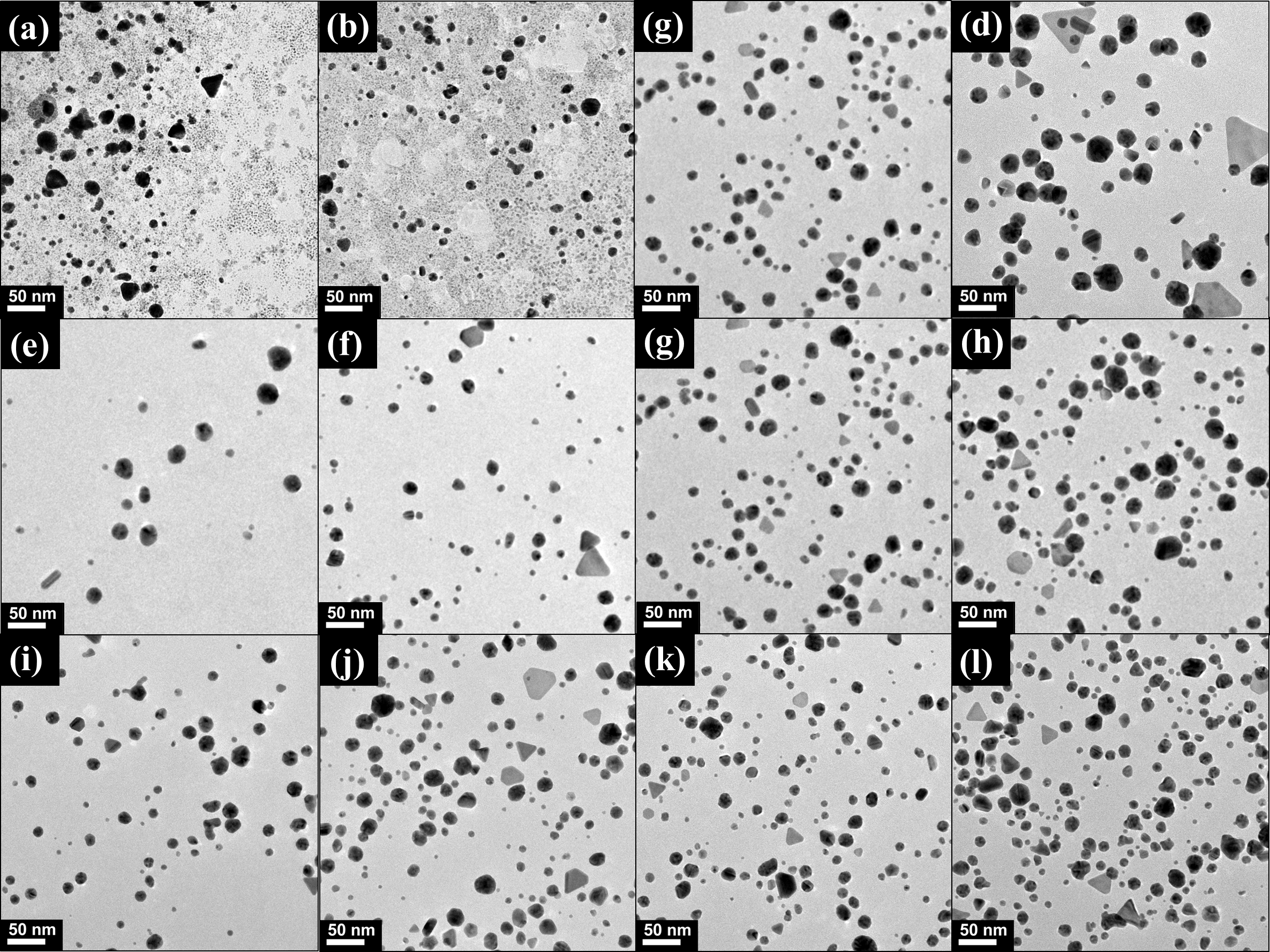
**

**Fig. S2**. TEM images of lignin-AuNP composites synthesized under different reaction conditions: (a) LigAu1, (b) LigAu2, (c) LigAu3, (d) LigAu4, (e) LigAu5, (f) LigAu6, (g) LigAu3, (h) LigAu7, (i) LigAu9, (j) LigAu10, (k) LigAu12 and (l) LigAu15.

Fig. S2(a)-(d) exhibit the TEM images of lignin-AuNP composites with different mass ratios of lignin to Au precursors. Fig. S2(a) and (b) shows AuNPs with unsatisfactory dispersion, and there were a large amount of unripe Au nanocrystals. It can be seen from Fig. S2(c) and (d) that the dispersity of AuNPs became much better, which is also shown in the UV-Vis result of Fig. 2(a). The TEM images of lignin-AuNP composites synthesized under different temperatures are shown in Fig. S2(e)-(h), illustrating that the amount of AuNPs increased with the increasing irradiation temperature, which is in agreement with the UV-Vis and elemental analysis results. Furthermore, it can be observed that the AuNPs were basically spherical, with a small portion of rods and plates, which may have been induced by relatively low pH at the very beginning of the reaction [2]. From Fig. S2(i)-(l), similar shapes and sizes of AuNPs can be seen with the prolonged reaction time, and the amount of nanoparticles gradually increased. These results are consistent with the UV-Vis result in Fig. 2(a) and the elemental analysis in Table S1.


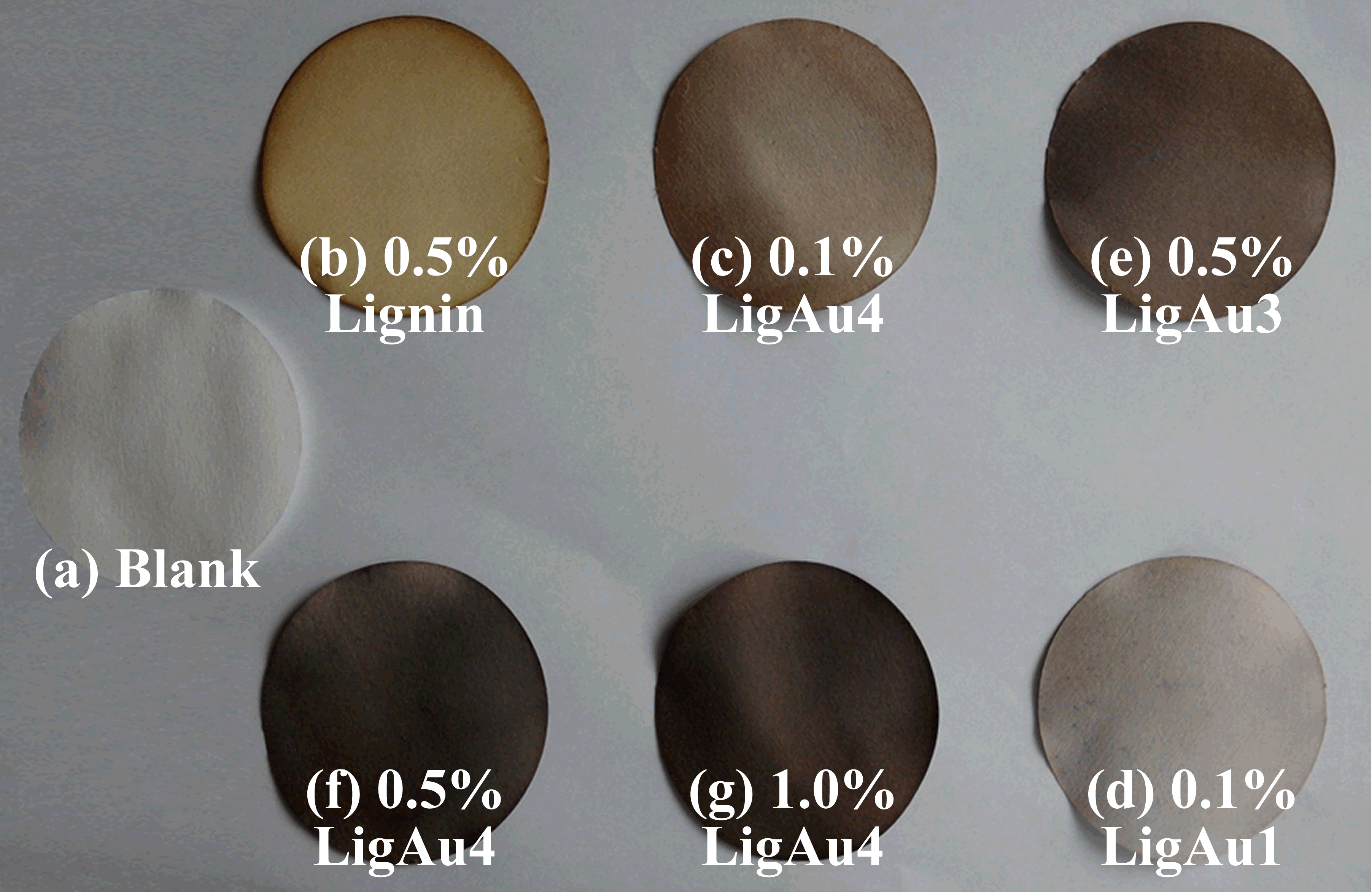


**Fig. S3**. Optical photo of lignin-AuNP composite papers with different concentrations and Au contents: (a) Blank, (b) 0.5% Lignin, (c) 0.1% LigAu4, (d) 0.5% LigAu1, (e) 0.5% LigAu3, (f) 0.5% LigAu4 and (g) 1.0% LigAu4.

As shown in Fig. S3, paper immersed with 0.5 % lignin looked yellowish (the natural color of lignin), and the color of paper immersed with different concentrations and contents of lignin-AuNP composites turned from grey to dark brown.


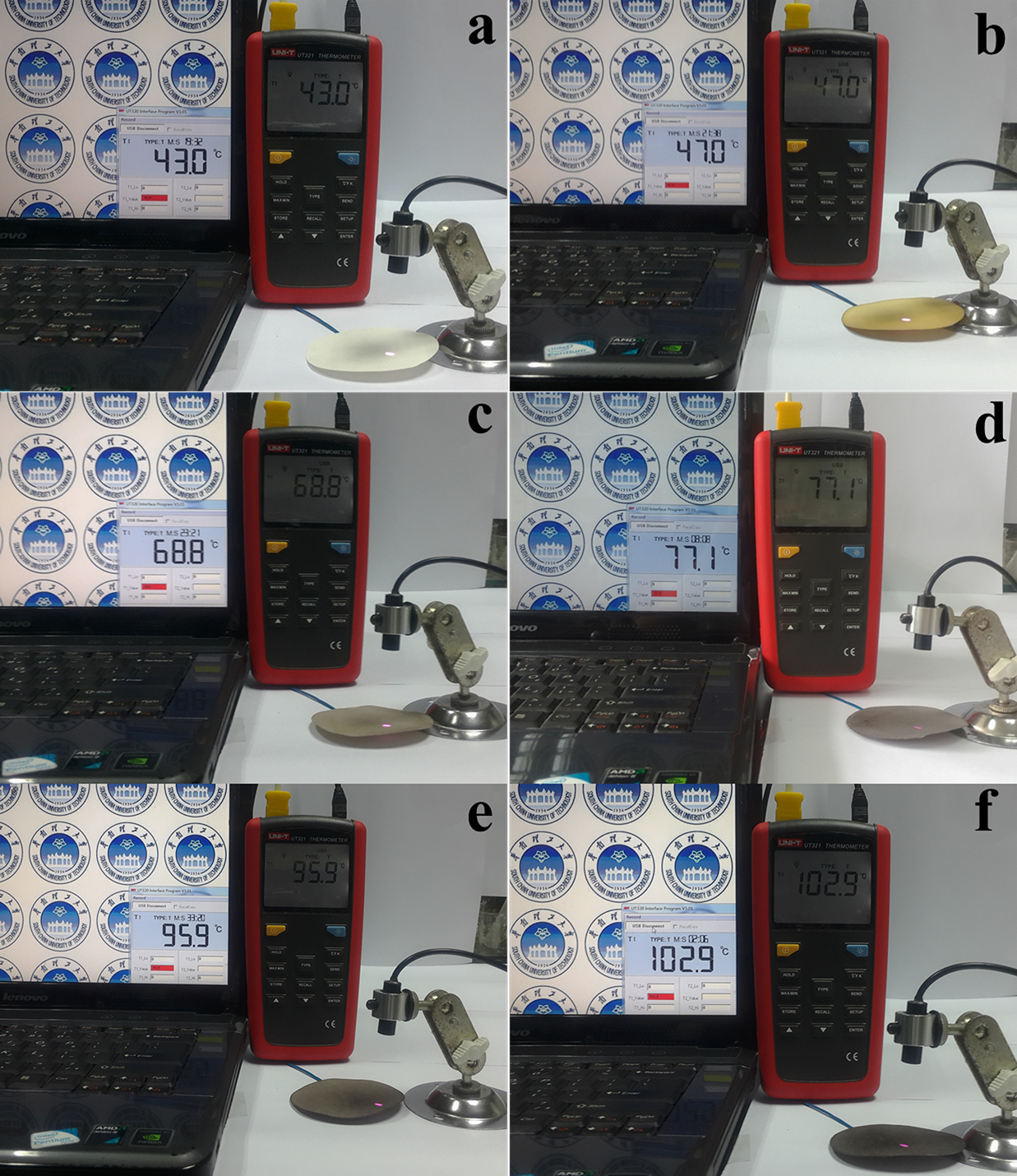


**Fig. S4**. Optical image of the ultimate temperature of lignin-AuNP composite papers: (a) Blank, (b) 0.5% Lignin, (c) 0.1% LigAu4, (d) 0.5% LigAu1, (e) 0.5% LigAu3 and (f) 0.5% LigAu4.

The temperature variation of the thermal probe with the prolonged irradiation time was recorded and plotted by a thermometer and computer software, and the average heating rate in the period of 60 s was calculated. The results are shown in Table S2 and Fig. S4. The blank sample showed little increment in temperature. The lignin-only paper performed a little better than blank sample. It can be clearly seen that paper immersed with lignin-AuNP composites exhibited a quicker temperature increment than the samples without AuNPs, which is positively proportional to the concentration of AuNPs.

The adsorption property of lignin-AuNPs liquid marbles towards Pb^2+^ under irradiation was studied, the whole experiment lasted a few minutes and was recorded in Movie S1.

Video S1. NIR-triggered detection and adsorption of lignin/AuNP liquid marble for Pb^2+^.

**References**

1. M. Garcia, J. De la Venta, P. Crespo, J. Llopis, S. Penadés, A. Fernández, A. Hernando, Surface plasmon resonance of capped Au nanoparticles, Phys. Rev. B 72 (2005) 241403.
2. X. Zhang, X. He, K. Wang, F. Ren, Z. Qin, pH induced protein-scaffold biosynthesis of tunable shape gold nanoparticles, Nanotechnology 22 (2011) 355603.
